# Pregnancy rate after endometrial injury in couples with unexplained infertility: A randomized clinical trial

**Published:** 2013-11

**Authors:** Mohammad Ebrahim Parsanezhad, Nasrin Dadras, Najmeh Maharlouei, Leila Neghahban, Peghah Keramati, Madihe Amini

**Affiliations:** 1 *Infertility Research Center, Shiraz University of Medical Sciences, Shiraz, Iran.*; 2 *Health Policy Research Center, Shiraz University of Medical Sciences, Shiraz, Iran.*

**Keywords:** *Infertility*, *Female*, *Endometrial injury*, *Pregnancy*

## Abstract

**Background:** Unexplained infertility is still a challenging issue as to its causes, appropriate management and treatment. Evidence implicates early embryopathy or implantation failure as likely causes.

**Objective: **This study aims to investigate the effect of local endometrial injury on pregnancy rate in selected unexplained infertile patients.

**Materials and Methods:** This was a randomized clinical trial conducted in Shiraz University Infertility Clinic of Ghadir Hospital. A total of 217 women with unexplained infertility aged 23-35 years old were randomly divided into two study groups through block randomization. After superovulation by clomiphene-citrate and gonadotropins and when the dominant follicles reached 18-20 mm, patients were randomly assigned to undergo endometrial local injury at posterior uterine wall by piplle endometrial sampling (n=114) or mock pipette biopsy (n=103) during pre-ovulatory days (when spontaneous urinary LH surge was detected). Then all the patients were instructed to follow a regularly timed intercourse.

**Results: **The pregnancy rate was significantly higher in the endometrial injury group compared to the control group [17/114 (14.9%) vs. 6/103 (5.8%) (OR: 2.83 95% CI: 1.07-7.49, p=0.03]. The abortion rate was comparable between two groups (17.64% vs. 14.28%; p=0.701).

**Conclusion:** Local mechanical injury of the endometrium can enhance the uterine receptivity and facilitates the embryo implantation. This simple, easy, and cost effective procedure is worth considering in selective unexplained infertility patients who implantation failure is the likely causes of infertility before complex treatments. This procedure may help reduce psychological tensions and high expenses imposed through such interventions.

**Registration ID in IRCT:** IRCT2012082510657N1

## Introduction

Traditionally, evaluation and treatment for infertility is usually postponed until the first year of natural attempts (1). In the women over 35, however the workup is preferred to be done after 6 months (2). Unexplained infertility (UI; no identifiable cause of infertility on a routine evaluation) may only reveal the lower extreme of normal fertility or limitation of the present diagnostic tests to identify a specific cause (3). 10-30% of couples are diagnosed with UI based on the diagnostic criteria (4). 

Evidence indicating that about 75% of human pregnancies fails soon after conception implicates early embryopathy or implantation failure as the possible causes of unexplained infertility (5). Interactions between endometrium and the embryo as well as endometrial receptivity are considered as two strong factors affecting the outcome of implantation. The endometrial receptivity is self-limited and is usually limited to the days 19-24 of the menstrual cycle in humans. Animal studies have demonstrated that scratching and trauma to the endometrium provoke the decidualization and, consequently, enhance the endometrial receptivity in animals (6). 

In this regard, Barash *et al *demonstrated that performing endometrial biopsy on days 8, 12, 21, and 26 of the menstrual cycle is associated with higher pregnancy rate after Invitro fertilization (IVF) (7). These results were further confirmed by Zhou *et al* who showed that inducing local injury to the endometrium in Controlled Ovarian Hyperstimulation (COH) cycles is associated with a higher success rate (8). However, Karimzade *et al* have shown that local injury to the endometrium on the day of oocyte retrieval disrupts the receptive endometrium and has a negative impact on implantation in IVF cycles (9).

Increasing age of the female partner and duration of infertility are the influential factors in the likelihood of pregnancy (10). Based on the findings of several studies, untreated patients have a cycle fecundity of about 2-4% as compared with 20-25% in normal fertile couples, showing about 80-90% reduction in the fecundity rate (11). Although it is reasonable to consider no intervention or expectant management therapy with younger unexplained infertile patients, treatment can apparently be more effective in couples with a longer duration of infertility who have a lower chance for conceiving without treatment (12). 

This randomized clinical trial aimed to investigate the effects of localized endometrial injury on the outcome of pregnancy in the patients suffering from UI.

## Materials and methods

The current prospective, randomized clinical trial was performed in one infertility clinic from January 2010 to March 2012. The study protocol was approved by the institutional review board (IRB) and Ethics Committee of Shiraz University of Medical Sciences and all the patients gave their informed written consents.

**Patient recruitment and treatment**

This study was conducted on 234 patients with unexplained infertility referring to our clinic during the study period. All the patients had normal ovulatory function, normal uterine cavity, and bilateral tubal patency via hystrosalpingography and/or hystrolaprascopy if indicated. Unexplained infertility was diagnosed after exclusion of all the known infertility etiologies such as hormonal disorders, infections, genetic anomalies, immunologic problems, and abnormal anatomic structures. 

All the included women were between 23 and 35 years of age, had an infertility duration of 2-5 years, body mass index (calculated as weight in kilograms divided by the height squared in meters) of 18-25 kg/m^2^, antimullerian hormone (AMH) more than 1 µg/l, follicle stimulating hormone (FSH) levels of less than 10 mIU/ml on the 3rd day of the cycle, and at least 10-12 follicles in antral follicle count (AFC). The women under study had only received clomiphene citrate for their infertility during the three past months and none of them had received gonadotropins or any other interventions for treatment of their infertility. 

All the male partners had normal semen analyses parameters (defined by the threshold values of the World Health Organization i.e., concentration of more than15×10^6^/mL, total count of 39×10^6^, progressive motility more than of 32%, and normal morphology of at least 4%) (13). There were no painters or factory workers among the males under study. In addition, none of the participants smoked or had a history of alcohol abuse. The study subjects were randomly assigned to two groups through block randomization. All the patients underwent the same optimal superovulation by clomiphene-citrate (Iran Hormone pharmaceutical Co.) orally administered 100mg/day from day 3 to 7 day of the cycle and HMG-Merional (IBSA, Lugarno, Switzerland) intramuscularly administered 75 UI/day from the 6^th^-8^th^ day. 

When the dominant follicles reached 18 mm, as measured by transvaginal ultrasound, an available commercial kit was used in order to finding the spontaneous urinary LH surge (twice/daily, 10 AM and 2-4 PM till LH surge was detected,). Mild endometrial local injury was performed in the posterior wall of the uterus by standard piplle endometrial sampling (Pipelle de Cornier, Prodimed, Neuilly-en-Thelle, France) during the preovulatory days (the day of detecting urinary LH-surge) just in the case group. 

Those who were randomly assigned to control group underwent gynecological examination using a mock pipelle biopsy without any endometrial manipulation (no entry of pipelle into internal os of cervix). Then, all the patients in both groups were instructed to follow a regular timed intercourse (from LH positive days until 8 days later every other day). β-hCG was checked if the patient experienced one week missed period. Moreover, pregnancy was documented by transvaginal sonography at 6-7 weeks of gestation. The main outcome measurements were pregnancy rate, abortion rate, and ongoing pregnancy rate (calculated by subtracting abortion from pregnancy rate).

**Statistical analysis**

Considering 80% power to detect the significant differences between main study outcome which was pregnancy rate (α=0.05, two-sided), 98 patients were needed in each study group. To compensate for the possible none valuable data, we enrolled 117 participants in each group. Independent-test was used to compare the results within the groups, and chi-square test was used to compare the proportions. The data are reported as mean±SD. Besides, p<0.05 was considered as statistically significant. The results were analyzed using SPSS statistical software (version 11.5) (SPSS Inc. Chicago IL). 

## Results

**Figure 1:**
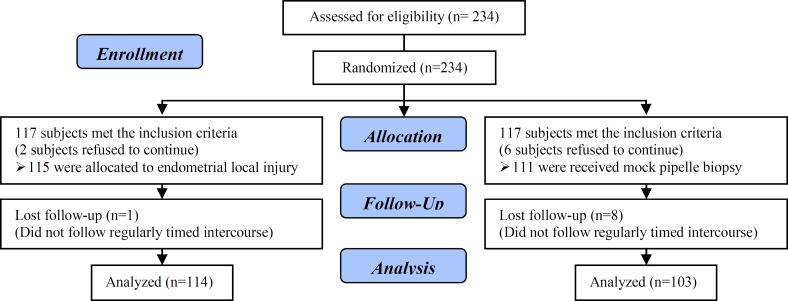
Consort statement flow diagram

Figure 1 shows the consort flowchart of the trial. A total of 234 eligible women were enrolled into the study and randomly divided into two study groups (117 each). Then, 8 out of the 234 women did not continue their participation in the study. Therefore, 226 unexplained infertile women aging between 23 and 35 years old continued their participation in the study. After optimal superovulation, 115 women underwent endometrial local injury and 111 women underwent mock pipell biopsy. 

One subject in the injury group and 8 patients in the control group did not follow the regularly timed intercourse and, consequently, the data of 114 patients in the injury group and 103 ones in the control group were analyzed. The baseline characteristics of the patients in two study groups are summarized in table I. There were no differences between the two study groups regarding the demographic characteristics, BMI, duration of infertility, basal FSH, AMH, duration and dose of hormone stimulation, endometrial thickness, and number of mature follicles of at least 18 mm. 

The study results revealed a significant difference between the two groups regarding pregnancy rate [20/114 (17.5%) vs. 7/103 (6.7%), p=0.027]. However, no significant difference was observed between the two groups regarding the abortion rate [3 (17.64%) vs. 1 (14.28%), p=0.701]*. *The ongoing pregnancy rate was significantly higher in the endometrial injury group compared to the control group [17/114 (14.9%) vs. 6/103 (5.8%) (OR: 2.83 95% CI: 1.07-7.49, p=0.03] (Table II).

**Table I:** Baseline clinical characteristics of the two groups

**Parameters**	**Injury group** ** (n=114)** **(Mean ± SD)**	**Control group (n=103)** **(Mean ± SD)**	**p-value**
Age (years)	30.0 ± 3	30.32 ± 2	0.299
BMI (kg/m^2^)	23.3 ± 2.4	23.7 ± 2.5	0.230
AMH (micrograms/liter)	2.50 ± 0.10	2.52 ± 0.13	0.203
Basal FSH (mIU/ml)	4.9 ± 1.2	5.2 ± 0.9	0.076
Duration of infertility	3.4 ± 1.1	3.6 ± 1.4	0.341
HMG ampoules per cycle	3.9 ± 0.7	4.1 ± 0.8	0.050
Endometrial thickness	8.94 ± 1.21	9.18 ± 1.3	0.162
Number of follicles ≥18mm	2.9 ± 0.7	2.8 ± 0.4	0.204

All the parameters in this table are expressed in mean± SD with student* t* test.

BMI=Body Mass Index

**Table II:** Results of pregnancy rate in both groups

**Parameters**	**Experimental group (n= 114)**	**Control group (n= 103)**	**p-value**
Pregnancy rate	20/114(17.5%)	7/103 (6.7%)	0.027*
Abortion rate	3 (17.64%)	1 (14.28%)	0.701
Ongoing pregnancy rate	17 (14.9%)	6 (5.8%)	0.03*

All the parameters in this table are expressed in frequency

(%) with Chi-square test.

* p-value less than 0.05 was considered statistically significant.

## Discussion

We found that in comparison to the control group, endometrial injury within the late follicular phase was associated with higher pregnancy rate. The endometrium is receptive to embryonic apposition, attachment, and invasion during a defined implantation window (during midsecratory days of the menstrual cycle). Endometrial local injury has been previously proved to increase the fertility rate following assisted reproductive technologies (ART), including IVF and Intra-cytoplasmic sperm Injection (ICSI), with conflicting results (7-9). In the present study, the patients receiving endometrial local injury had higher pregnancy rate compared to the control group (17.5% vs. 6.7%, p=0.027).

Nonetheless, no significant difference was found between the two groups concerning the abortion rate (p=0.701). These results are in agreement with those of the study by Barash *et al* (7). They included 134 patients, defined as good responders to hormonal stimulation, who failed to conceive during one or more cycles of IVF and Embryo transfer (ET). The IVF and ET treatments were preceded by repeated endometrial biopsies in 45 participants. Their results suggested that the IVF treatment preceded by endometrial biopsy doubled the chance for a take-home baby. Another similar study was done by Zouh *et al* who also investigated the possibility that local injury to the endometrium in COH cycle improves the incidence of embryo implantation in IVF-ET (8). 

They included 121 infertile women undergoing endometrial biopsy or nothing before IVF-ET and found that local injury to the endometrium during a COH cycle improved the rates of embryo implantation, clinical pregnancy, and live birth in ART. In a similar study, Gnainsky *et al* found that a biopsy-induced inflammatory response may facilitate the preparation of the endometrium for implantation (14). They suggested that increased MIP-1B expression could possibly serve for prediction of implantation competence. However, conflicting results were reported by Karimzade and colleagues (9). 

They evaluated the effect of local injury to the endometrium on the day of oocyte retrieval on implantation and pregnancy rates in assisted reproductive cycles. The results demonstrated that local injury to the endometrium on the day of oocyte retrieval disrupted the receptive endometrium and had a negative impact on implantation in IVF cycles. We performed endometrial injury in the late follicular phase in order to ensure having sufficient time for cytokine production, gene expression, and other positive effects of the injury on the endometrium until the implantation days. In some previous studies trauma to the endometrium was done in the luteal phase preceding the COH, while it was performed in both follicular and lutheal phases in some others (7-9). 

Although up to now, luteal phase induced endometrial injury has been reported to be associated with the highest decidualization, there are still many unanswered questions regarding patient selection, timing, technique, and number of required biopsies. Thus, further studies are needed in order to answer these questions. There are some possible suggested mechanisms by which the endometrial sampling may increase receptivity and improve clinical pregnancy rate. The first one is decidualization of endometrium. As it was mentioned by Barash *et al* Loeb was pioneer in reporting that scratching guinea-pig uteruses provoked the rapid growth of the endometrial cells which are similar to the decidual cells of pregnancy (7).

The next mechanism is wound healing process involving a massive secretion of different cytokines and growth factors which are beneficial for embryo implantation (15). Also, the last mechanism is synchronization of endometrial and embryo development. Mirkin *et al* reported that COH cycles resulted in different structural and functional changes in comparison to natural cycles, including histological advancement, pinopodes maturation advancement, and steroid receptor down-regulation (16). Unexplained infertility is still a challenging issue although there is no consensus about its definition and treatment. Moreover, the results obtained from different studies conducted on the issue are not in agreement since they have been performed on heterogeneous populations (17). 

When counseling the patients regarding the treatment options, both the expected in cycle fecundity and the treatment cost should be taken into account. Typical interventions proceed stepwise with super ovulation (first with clomiphene or letrazole for three to four cycles, then with gonadotropins for three to four cycles) combined with intrauterine insemination followed by ART(18). One of the advantages of this study was that we tried to follow the patients’ natural cycle rather than the routine interventions, such as IUI or regular administration of adjuant HCG for which no logic has been considered. In the study by Kosmas *et al*, injection of HCG led to the exacerbation of the results and imposing more cost and inconvenience on the patients. It may also increase the likelihood of atresia of other follicles which were beneficial to pregnancy (19). 

We note some limitations to our study. First, not only the majority of our study patients were young with short durations of infertility, but they also had desirable ovarian function which was presented by their acceptable levels of FSH, AMH, and AFC. Therefore, they could not represent the general UI population and, consequently, the results cannot be generalized to the general UI patients. Of course, this can be in a way considered as a strong point of this study because it shows that injury is mostly beneficial for the UI patients whose infertility is most likely due to implantation failure. 

Second, the pregnancy rate of our study patients was low because due to the fact that we wanted to determine the role of endometrial injury alone, we used timed intercourse instead of IUI as the fertility inducing intervention. Third we did not measure the inflammatory markers of endometrium, so that we cannot comment on role of inflammation on implantation. Further studies are required to elucidate the effects of endometrial injury on the outcome of patients with UI. 

## Conclusion

The study findings revealed that endometrial local injury could be considered as one of the interventions which increased endometrial receptivity in UI patients whose infertility was not suspicious of egg problem. Therefore, if the results of the present study are confirmed by further studies on other populations, endometrial local injury could be considered as one of the treatment methods for selected UI couples whose infertility most likely due to implantation failure. This simple, easy, and cost effective procedure is worth considering in infertile couples especially in younger couples with shorter duration of infertility before complex treatments. This procedure may help reduce psychological tensions and high expenses imposed through such interventions.
